# Evolution of Amoxicillin-Based Mono-Antibiotic Regimens for *Helicobacter pylori* Eradication: From Ineffectiveness to Innovation—A Systematic Review

**DOI:** 10.3390/microorganisms14030625

**Published:** 2026-03-10

**Authors:** Chih-An Shih, I-Ting Wu, Deng-Chyang Wu, Wei-Yi Lei, Feng-Woei Tsay, Tzung-Jiun Tsai, Chung-Hung Tsai, Ya-Chi Wu, Wei-Chen Tai, Ping-I Hsu

**Affiliations:** 1 Division of Gastroenterology and Hepatology, Department of Internal Medicine, Antai Medical Care Corporation, Antai Tian-Sheng Memorial Hospital, Pingtung 928, Taiwan; dreric921@gmail.com; 2 Department of Nursing, Meiho University, Pingtung 912, Taiwan; 3 Division of Gastroenterology, Department of Internal Medicine, An Nan Hospital, China Medical University, Tainan 709, Taiwan; ilessalen@gmail.com; 4 Division of Gastroenterology, Department of Internal Medicine, Kaohsiung Medical University Hospital, Kaohsiung Medical University, Kaohsiung 807, Taiwan; 5 Department of Medicine, Buddhist Tzu Chi General Hospital, Tzu Chi University, Hualien 970, Taiwan; 6 Division of Gastroenterology and Hepatology, Department of Internal Medicine, Kaohsiung Veterans General Hospital, National Yang Ming Chiao Tung University, Kaohsiung 813, Taiwan; 7 Department of Family Medicine, An Nan Hospital, China Medical University, Tainan 709, Taiwan; 8 Division of Gastroenterology, Department of Internal Medicine, Kaohsiung Chang Gung Memorial Hospital, Chang Gung University College of Medicine, Kaohsiung 833, Taiwan

**Keywords:** *Helicobacter pylori*, amoxicillin, bismuth, potassium competitive acid blocker (PCAB), dual therapy, triple therapy, eradication therapy, antibiotic resistance

## Abstract

Mono-antibiotic therapy for *Helicobacter pylori* (*H. pylori*) infection minimizes unnecessary antibiotic exposure, reduces disruption of the gut microbiota, and lowers the risk of multidrug resistance. Although resistance of *H. pylori* to amoxicillin remains extremely low (<3%) worldwide, regular-dose amoxicillin monotherapy achieves eradication rates of less than 30%. Strategies to improve the efficacy of amoxicillin-based mono-antibiotic therapy include elevating intragastric pH with potent acid suppression, increasing the amoxicillin dose, and adding bismuth salts to the treatment regimen. This review evaluates the safety and effectiveness of six amoxicillin-based treatments for *H. pylori*. All regimens lasted 14 days and were studied in clinical trials published between 1 October 2014, and 1 October 2025. The pooled intention-to-treat and per-protocol eradication rates for each regimen were as follows: Regimen 1: Regular-dose amoxicillin + high-dose proton pump inhibitor (PPI): 84.7% (83/98) and 84.7% (83/98); Regimen 2: High-dose amoxicillin + high-dose PPI: 85.3% (3709/4347) and 89.9% (3692/4109); Regimen 3: Regular-dose amoxicillin + high-dose potassium-competitive acid blocker (PCAB): 86.0% (901/1048) and 91.2% (888/974); Regimen 4: High-dose amoxicillin + high-dose PCAB: 88.2% (1771/2009) and 93.5% (1720/1839); Regimen 5: Regular-dose amoxicillin + high-dose PCAB + bismuth: 84.9% (327/385) and 91.3% (327/358); Regimen 6: High-dose amoxicillin + high-dose PCAB + bismuth: 95.8% (115/120) and 98.4% (115/117). In conclusion, potent acid inhibition, escalation of amoxicillin dosage, and incorporation of bismuth can transform amoxicillin mono-antibiotic therapy from an ineffective approach into a highly effective eradication regimen for *H. pylori* infection.

## 1. Introduction

*Helicobacter pylori* (*H. pylori*) was previously known as *Campylobacter pylori*. The name change occurred after detailed analysis determined that the organism fit better in the new genus *Helicobacter*, rather than *Campylobacter*, due to its distinct characteristics.

*H. pylori* remains the principal etiological factor underlying chronic gastritis, peptic ulcer disease—including both gastric and duodenal ulcers—as well as gastric adenocarcinoma and mucosa-associated lymphoid tissue (MALT) lymphoma [1,2,3]. Current international recommendations advocate offering eradication therapy to all patients in whom *H. pylori* infection has been confirmed [4,5]. The success of eradication therapy is strongly influenced by antimicrobial susceptibility. In recent years, primary resistance of *H. pylori* to clarithromycin, metronidazole, and levofloxacin has risen substantially worldwide [6,7]. Contemporary surveillance studies report resistance rates ranging from 8–67% for clarithromycin, 35–74% for metronidazole, and 21–43% for levofloxacin, whereas resistance to amoxicillin (0–8%) and tetracycline (0–4%) remains comparatively low [6]. The marked increase in clarithromycin resistance has significantly compromised the efficacy of conventional triple therapy, with eradication rates falling below 80% in many regions. According to the Kyoto Global Consensus on *H. pylori* gastritis, empirical regimens should only be adopted if they consistently achieve eradication rates of at least 90% at the population level [8]. To meet this benchmark, several alternative first-line strategies have been developed, including bismuth-containing quadruple therapy and non-bismuth quadruple regimens such as sequential, concomitant, and hybrid therapies [9,10,11].

Although many international guidelines endorse bismuth quadruple therapy (BQT) as the preferred first-line option in settings with elevated clarithromycin resistance [9,10,11], its clinical application is not without drawbacks. BQT involves a relatively complicated dosing schedule and has been associated with a high incidence of treatment-related adverse events in large randomized controlled trials [12,13,14,15]. In two large-scales randomized controlled trials, nearly half to more than half of treated patients (48% and 56%, respectively) experienced adverse effects [14,15]. Commonly reported events include dysgeusia, tongue discoloration, darkened stools, nausea, and abdominal discomfort [16,17,18,19,20].

Clarithromycin-based non-bismuth quadruple regimens, particularly concomitant therapy, have been shown to maintain favorable eradication outcomes even in the presence of clarithromycin resistance [11]. Concomitant therapy consists of four agents—a proton pump inhibitor (PPI), clarithromycin, amoxicillin, and metronidazole—administered simultaneously throughout the entire treatment period. Evidence from meta-analytic data indicates that this regimen provides superior eradication rates compared with conventional standard triple therapy. Hybrid therapy, a modified clarithromycin-containing quadruple regimen developed by our group, represents another non-bismuth strategy [9]. This approach begins with 7 days of dual therapy comprising a PPI plus amoxicillin, followed by 7 days of quadruple therapy including a PPI, amoxicillin, clarithromycin, and metronidazole. In an initial pilot investigation conducted in Taiwan, hybrid therapy achieved eradication rates of 97.4% in the intention-to-treat (ITT) population and 99.1% in the per-protocol (PP) analysis [9]. Subsequently, a large multicenter randomized controlled trial demonstrated that 14-day hybrid therapy achieved efficacy comparable to 14-day BQT, with both regimens curing over 90% of patients in regions where clarithromycin resistance was moderate (approximately 17%) [15]. Notably, the incidence of adverse events was lower with hybrid therapy than with BQT. Despite its effectiveness, hybrid therapy requires the addition of two extra antibiotics during the second treatment phase, which may complicate dosing schedules and reduce patient acceptance in routine practice. To address this limitation, the treatment sequence was reversed—initiating therapy with a quadruple regimen followed by a dual regimen—thereby streamlining the medication schedule. This “reverse hybrid” strategy avoids introducing new drugs midway through therapy. A multicenter randomized trial confirmed that 14-day reverse hybrid therapy produced eradication rates similar to those achieved with 14-day BQT, while also demonstrating a more favorable safety profile [14]. Nevertheless, whether in the form of BQT, concomitant therapy, hybrid therapy, or reverse hybrid therapy, these regimens share a common characteristic: eradication relies on the use of two to three antibiotics.

Currently, the resistance rates to amoxicillin and tetracycline remain extremely low (<3%) in most countries [21,22]. Amoxicillin alone as mono-antibiotic therapy without an anti-acid agent has been used since 1988 for first-line anti-*H. pylori* treatment. Despite being treated with oral anti-*H. pylori* low-dose amoxicillin-alone monotherapy, amoxicillin by itself achieves less than a 30% eradication rate of *H. pylori* in Table 1 [23,24,25]. The clinical trial study showed 22 *H. pylori*-infected patients that received amoxicillin 375 mg treatment three times a day for 4 weeks. Eradication of *H. pylori* on culture was reduced from 45% immediately after treatment to 23% 4 weeks later in the amoxicillin-alone group [23]. Another clinical trial study showed 42 pediatric patients received amoxicillin 25 mg/kg twice daily for 4 weeks. Amoxicillin alone does not seem to be able to eradicate *H. pylori* in the long term, and its relapse rate is unacceptably high (73%) [25].

Strategies to improve the efficacy of amoxicillin-based mono-antibiotic therapy include elevating intragastric pH with potent acid suppression, increasing the amoxicillin dose, and adding bismuth salts to the treatment regimen. Elevating intragastric pH above 5.0 induces *H. pylori* to shift into a replicative state, thereby enhancing *H. pylori* susceptibility to amoxicillin [25]. Experimental data have shown that the antibacterial activity of amoxicillin against *H. pylori* is strongly influenced by intragastric pH. In vitro findings indicate that when the surrounding pH rises from 5.5 to 7.5, the minimal inhibitory concentration required to inhibit 90% of isolates (MIC_90_) declines markedly, from 0.5 mg/L to 0.06 mg/L [25]. These results suggest that optimizing acid suppression may significantly enhance the bactericidal effect of amoxicillin. Administration of high-dose proton pump inhibitors (PPIs) is capable of sustaining intragastric pH levels above 6, independent of *CYP2C19* metabolic polymorphisms, thereby improving the antimicrobial effectiveness of amoxicillin [26]. Clinical evidence supports this pharmacologic rationale. In a randomized controlled study conducted in Taiwan, dual therapy consisting of high-dose amoxicillin combined with rabeprazole achieved an ITT eradication rate of 95%, which was superior to that observed with conventional triple therapy (81%) [27]. Beyond its favorable eradication performance, this dual regimen offers several practical advantages, including simplified dosing, a lower incidence of adverse events, and reduced exposure to additional antibiotics. Nevertheless, accumulating international data indicate that the therapeutic success of high-dose amoxicillin–PPI dual therapy is not uniform worldwide and appears to vary by geographic region.

Vonoprazan is a next-generation acid suppressant classified as a potassium-competitive acid blocker (P-CAB) [28,29]. Unlike conventional PPIs, it suppresses gastric acid secretion through reversible inhibition of the gastric H^+^/K^+^-ATPase. Pharmacodynamic studies have demonstrated that vonoprazan provides more potent and sustained acid suppression compared with standard PPIs. This enhanced and stable elevation of intragastric pH may improve the effectiveness of *H. pylori* eradication therapy by creating a more favorable environment for antibiotic activity. In terms of metabolism, vonoprazan is primarily processed in the liver via cytochrome P450 CYP3A4, with minor contributions from SULT2A1, CYP2C19, CYP2B6, and CYP2D6. Multiple randomized controlled trials as well as observational studies have consistently reported that a 7-day vonoprazan-based triple regimen achieves higher eradication rates than a 7-day PPI-based triple regimen when used as first-line therapy for *H. pylori* infection [28,29]. Furthermore, clinical studies suggest that vonoprazan combined with amoxicillin as dual therapy can yield eradication outcomes comparable to those of vonoprazan-containing triple therapy [26]. Another strategy to further optimize treatment efficacy involves the addition of bismuth. Bismuth compounds contribute to eradication through multiple mechanisms, including disruption of bacterial cell wall integrity and interference with mucosal adherence. In addition, bismuth suppresses key enzymatic functions of *H. pylori*, such as urease, phosphatase, and protease activities, thereby enhancing antibacterial effects [29].

Mono-antibiotic therapy for *H. pylori* infection minimizes unnecessary antibiotic exposure, reduces disruption of the gut microbiota, and lowers the risk of multidrug resistance. This review article aims to assess the effectiveness and safety of six 14-day amoxicillin-based regimens: (1) regular-dose amoxicillin with high-dose PPI, (2) high-dose amoxicillin with high-dose PPI, (3) regular-dose amoxicillin with high-dose PCAB, (4) high-dose amoxicillin with high-dose PCAB, (5) regular-dose amoxicillin with high-dose PCAB plus bismuth, and (6) high-dose amoxicillin with high-dose PCAB plus bismuth. In this study, high-dose amoxicillin was defined as dosage of amoxicillin ≥ 3 g/day.

## 2. Article Search

This systematic review was performed in accordance with the PRISMA (Preferred Reporting Items for Systematic Reviews and Meta-Analyses) statement to ensure methodological transparency and structured reporting [30]. The study protocol was prospectively registered in the PROSPERO database (registration number: CRD420261284583). The primary research question was defined as follows: among treatment-naïve patients with *Helicobacter pylori* infection, can 14-day amoxicillin-based mono-antibiotic regimens administered at regular or high doses achieve a per-protocol eradication rate of at least 90% when used as first-line therapy? A comprehensive literature search was conducted using PubMed, Embase, and the Cochrane Library. Search strategies combined controlled vocabulary and keywords related to “*Helicobacter pylori*” and “therapy” or “treatment,” targeting titles and abstracts. In addition to electronic database searches, manual screening of original research presented at major international conferences—including Digestive Disease Week (DDW), United European Gastroenterology Week (UEGW), Asia Pacific Digestive Disease Week (APDW), and meetings of the European Helicobacter Study Group—was undertaken. Eligible studies were restricted to English-language clinical trials published between 1 October 2014 and 1 October 2025 (Supplementary Materials).

Studies were considered eligible if they met the following criteria: (1) adult participants (≥18 years) receiving 14-day amoxicillin-based dual therapy with either a PPI or vonoprazan, or 14-day amoxicillin/vonoprazan/bismuth triple therapy as initial treatment for *H. pylori* infection; (2) randomized controlled trial design; (3) clearly reported eradication outcomes; and (4) publication in English. Exclusion criteria were: treatment duration shorter than 14 days; absence of confirmed *H. pylori* diagnosis before and after therapy using at least one validated diagnostic method (rapid urease test, histology, culture, or urea breath test); post-treatment assessment performed within four weeks of therapy completion; failure to report both ITT and PP results; publication solely as conference abstracts; or evaluation of rescue/salvage regimens.

**Figure 1:**
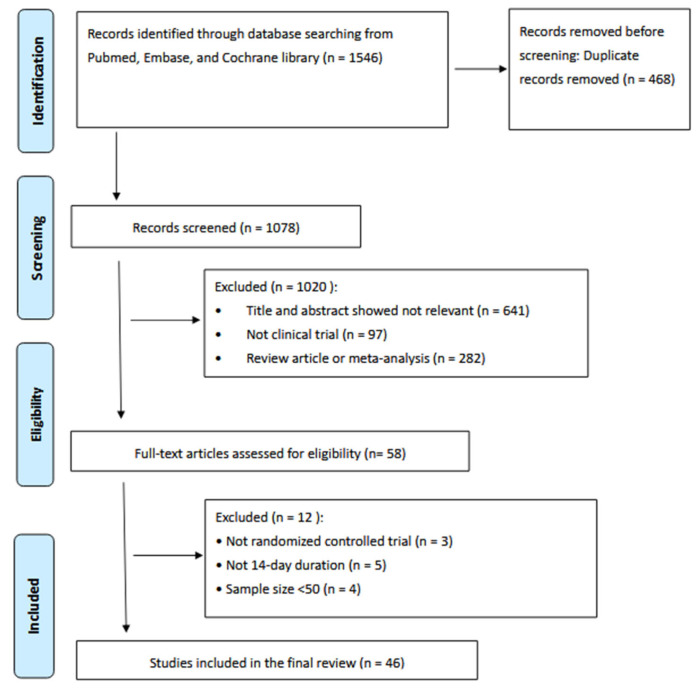
PRISMA diagram of the literature search.

Data extraction was conducted independently by two reviewers (C.A.S. and W.Y.L.) using a standardized collection form. Discrepancies were resolved through discussion with a third investigator (P.I.H.). Extracted variables included first author, year of publication, country of study, sample size, and eradication rates according to ITT and PP analyses. The initial search identified 1546 records. After removal of clearly irrelevant reports, reviews, meta-analyses, and non-clinical trial publications (*n* = 1488), the remaining studies underwent title and abstract screening. One duplicate record was excluded, resulting in 49 articles eligible for full-text review. Application of the predefined inclusion and exclusion criteria yielded 46 studies for final analysis. Statistical analyses were performed using MedCalc version 23.0.2 (MedCalc Software Ltd., Ostend, Belgium). The study selection process is illustrated in Figure 1.

### 2.1. Dual Therapies with Regular-Dose Amoxicillin/High-Dose PPI and High-Dose Amoxicillin/High-Dose PPI in the First-Line Treatment of H. pylori Infection

Table 2 presents the ITT and PP eradication outcomes of 14-day dual regimens combining regular- or high-dose amoxicillin with high-dose PPIs for first-line *H. pylori* therapy, based on randomized controlled trials published between 2014 and 2025 [16,17,18,19,20,27,31,32,33,34,35,36,37,38,39,40,41,42,43,44,45,46,47]. When data were pooled, the overall ITT eradication rates were 84.7% for regular-dose and 85.3% for high-dose amoxicillin regimens. Corresponding PP eradication rates were 84.7% and 89.9%, respectively. These findings indicate considerable geographic variability and demonstrate that such regimens do not reliably achieve the ≥90% eradication benchmark across different populations. Marked regional differences were evident. For example, a 14-day schedule consisting of amoxicillin 750 mg administered four times daily plus esomeprazole 40 mg three times daily achieved a PP eradication rate of 95.7% in a Taiwanese cohort [33]. In contrast, an identical dosing regimen evaluated in China resulted in a substantially lower PP eradication rate of 71.0% [42].

Several factors may account for the heterogeneity in treatment response observed with high-dose amoxicillin/PPI dual therapy. These include variations in local amoxicillin resistance rates, differences in the type and dosage of PPIs administered, genetic polymorphisms influencing PPI metabolism, medication adherence, body surface area (BSA), and dietary habits such as the intake of acidic foods. In a large multicenter randomized trial conducted by our group, both amoxicillin resistance and suboptimal adherence independently predicted treatment failure in patients receiving high-dose amoxicillin plus rabeprazole, with odds ratios of 8.2 and 8.6, respectively [20]. Within that study, eradication success was significantly reduced among patients harboring amoxicillin-resistant strains compared with those infected by susceptible strains (50% vs. 88%). Likewise, individuals with poor compliance demonstrated lower eradication rates than those who adhered well to therapy (40% vs. 87%). In addition, a separate Chinese study identified a BSA ≥ 1.69 m^2^ as the sole independent factor associated with eradication failure in high-dose amoxicillin/PPI dual therapy [48].

Regarding safety, pooled analyses showed that adverse events occurred in 13.3% (13/98) of patients receiving regular-dose regimens and 12.1% (517/4283) of those treated with high-dose regimens. Reported side effects were generally mild and most commonly included abdominal bloating, diarrhea, and nausea.

### 2.2. Dual Therapies with Regular-Dose Amoxicillin/High-Dose Vonoprazan and High-Dose Amoxicillin/High-Dose Vonoprazan in the First-Line Treatment of H. pylori Infection

Table 2 summarizes eradication outcomes from randomized controlled trials published between 2014 and 2025 evaluating 14-day dual regimens that combined vonoprazan with either regular- or high-dose amoxicillin as first-line therapy for *H. pylori* infection [47,49,50,51,52,53,54,55,56,57,58,59,60,61,62,63]. When pooled, ITT eradication rates were 86.0% for regular-dose and 88.2% for high-dose amoxicillin regimens. Corresponding PP eradication rates reached 91.2% and 93.5%, respectively. Despite these generally favorable results, treatment performance was not uniform across regions and did not consistently meet the ≥90% target in all populations. In China, most randomized trials reported PP eradication rates exceeding 90% with high-dose amoxicillin/vonoprazan dual therapy. However, studies conducted in the United States and Taiwan demonstrated lower PP eradication rates of 81.2% [55] and 87.2% [64], respectively. These findings indicate that further optimization is required before this regimen can be universally recommended as a standard first-line approach.

At present, determinants of treatment failure with 14-day high-dose amoxicillin/vonoprazan dual therapy are not fully defined. A Taiwanese randomized controlled trial identified suboptimal medication adherence as an independent predictor of eradication failure (odds ratio 8.4) [64]. In that study, patients with poor compliance achieved a markedly lower eradication rate compared with those who adhered well to therapy (60.0% vs. 87.7%). Additionally, a Japanese clinical investigation reported that larger BSA was associated with reduced efficacy in patients receiving 7-day standard-dose vonoprazan dual therapy (vonoprazan twice daily plus amoxicillin 750 mg twice daily) [65]. Similarly, diminished eradication success with high-dose vonoprazan dual therapy was observed among individuals with higher BSA (79.6% for BSA ≥ 1.723 m^2^ vs. 90.8% for BSA < 1.723 m^2^).

Regarding safety, pooled adverse event rates were 17.4% (201/1152) for regular-dose therapy and 19.8% (417/2108) for high-dose amoxicillin/vonoprazan dual therapy. Importantly, no serious treatment-related complications were documented in association with high-dose regimens. Reported adverse events were generally mild and included nausea, diarrhea, and abdominal bloating. Notably, two large studies by Chey et al. [55] and Cheung et al. [61] described overall adverse event rates of 29.9% and 39%, respectively, with diarrhea being the most commonly reported symptom (5.2% and 12.0%, respectively).

**Table 2:** Eradication rates of 14-day regular-dose amoxicillin/high-dose PPI dual, high-dose amoxicillin/high-dose PPI dual, regular-dose amoxicillin/high-dose vonoprazan dual, high-dose amoxicillin/high-dose vonoprazan dual, bismuth/regular-dose amoxicillin/high-dose vonoprazan triple and bismuth/high-dose amoxicillin/high-dose vonoprazan triple for the first-line treatment of *H. pylori* infection in randomized control trials conducted between 2014 and 2025.

14-Day Regular-Dose Amoxicillin/High-Dose PPI Dual Therapy and High-Dose Amoxicillin/High-Dose PPI Dual Therapy							
Author [Year]	Country	No. of Cases		Regimen	Eradication Rate		Adverse Events
					ITT	PP	
***Regular-dose amoxicillin*** Sapmaz et al. [31] [2017]	Turkey	98		rabeprazole 20 mg tid, amoxicillin 750 mg tid	84.7% (83/98)	84.7% (83/98)	13.3% (13/98)
All					84.7% (83/98)	84.7% (83/98)	13.3% (13/98)
***High-dose amoxicillin*** Yang et al. [27] [2015]	Taiwan	150		rabeprazole 20 mg qid, amoxicillin 750 mg qid	95.3% (143/150)	96.6% (143/148)	23.0% (34/148)
Hu et al. [32] [2017]	China	87		rabeprazole 20 mg qid, amoxicillin 750 mg qid	81.6% (71/87)	83.5% (71/85)	3.4% (3/87)
Tai et al. [33] [2019]	Taiwan	120		esomeprazole 40 mg tid, amoxicillin 750 mg qid	91.7% (110/120)	95.7% (110/115)	9.6% (11/115)
Yang et al. [16] [2019]	China	116		esomeprazole 20 mg qid, amoxicillin 750 mg qid	87.9% (102/116)	91.1% (102/112)	6.3% (7/112)
Yu et al. [34] [2019]	China	80		esomeprazole 40 mg bid, amoxicillin 1000 mg tid	92.5% (74/80)	96.1% (73/76)	7.5% (6/80)
Song et al. [35] [2020]	China	380		esomeprazole 20 mg qid, amoxicillin 750 mg qid	87.1% (331/380)	92.4% (329/356)	17.6% (66/375)
Zhang et al. [36] [2020]	China	104		esomeprazole 20 mg tid, amoxicillin 1000 mg tid	83.5% (76/91)	86.4% (76/88)	5.0% (5/101)
Hwong-Ruey et al. [37] [2020]	Malaysia	97		rabeprazole 20 mg qid, amoxicillin 1000 mg qid	92.8% (90/97)	93.8% (90/96)	20.5% (20/97)
Shen et al. [38] [2022]	China	496		esomeprazole 20 mg qid, amoxicillin 750 mg qid	88.31% (438/496)	91.63% (438/478)	13.3% (66/496)
Guan et al. [17] [2022]	China	350		esomeprazole 20 mg qid, amoxicillin 1000 mg tid	89.4% (313/350)	90.6% (308/340)	12.9% (45/349)
Han et al. [39] [2022]	China	315		esomeprazole 20 mg qid, amoxicillin 750 mg qid	88.6% (279/315)	90.4% (274/303)	13.7% (43/314)
Shao et al. [18] [2022]	China	120		rabeprazole 20 mg tid, amoxicillin 1000 mg tid	85.8% (103/120)	89.6% (103/115)	13.0% (15/115)
Bi et al. [40] [2022]	China	329		esomeprazole 40 mg tid, amoxicillin 1000 mg tid	75.4% (248/329)	81.3% (248/305)	11.1% (34/305)
Liu et al. [19] [2023]	China	422		esomeprazole 20 mg qid, amoxicillin 1000 mg tid	90.3% (381/422)	93.6% (381/407)	13.5% (55/407)
Hsu et al. [20] [2023]	Taiwan	306		rabeprazole 20 mg qid, amoxicillin 750 mg qid	83% (255/306)	87% (253/291)	13.0% (40/305)
Ding et al. [41] [2023]	China	134		esomeprazole 40 mg bid, amoxicillin 1000 mg tid	73.1% (98/134)	83.1% (98/118)	6.0% (8/134)
Yun et al. [42] [2023]	China	108		esomeprazole 40 mg tid, amoxicillin 750 mg qid	65.7% (71/108)	71.0% (71/100)	2.0% (2/100)
Zhang et al. [43] [2023]	China	101		ilaprazole 5 mg bid, amoxicillin 1000 mg tid	92.1% (93/101)	94.9% (93/98)	13.9% (14/101)
Macedo et al. [44] [2023]	Portugal	50		esomeprazole 40 mg bid, amoxicillin 1000 mg alternating with amoxicillin 500 mg qid	96.2% (48/50)	95.9% (47/49)	2.0% (1/50)
Valizadeh et al. [45] [2024]	Iran	114		esomeprazole 40 mg bid, amoxicillin 1000 mg tid	76.3% (87/114)	79.1% (87/110)	12.2% (14/114)
Han et al. [46] [2025]	China	160		ilaprazole 10 mg bid, amoxicillin 1000 mg tid	88.7% (142/160)	92.2% (142/154)	10.8% (17/157)
Yang et al. [47] [2025]	China	221		esomeprazole 40 mg qid, amoxicillin 750 mg qid	70.59% (156/221)	93.94% (155/165)	4.98% (11/221)
All					85.3% (3709/4347)	89.9% (3692/4109)	12.1% (517/4283)
**14-day regular-dose amoxicillin/high-dose vonoprazan dual therapy and high-dose amoxicillin/high-dose vonoprazan dual therapy**							
***Regular-dose amoxicillin*** Zuberi et al. [49] [2022]	Pakistan	96		vonoprazan 20 mg bid, amoxicillin 1000 mg bid	89.6% (86/96)	93.5% (86/92)	13.0% (12/92)
Hu et al. [50] [2023]	China	55		vonoprazan 20 mg bid, amoxicillin 1000 mg bid	89.1% (49/55)	94.1% (48/51)	29.1% (16/55)
Hu et al. [51] 2024]	China	95		vonoprazan 20 mg bid, amoxicillin 1000 mg bid	87.4% (83/95)	96.5% (83/86)	27.4% (26/95)
Liu et al. [52] [2024]	China	64		vonoprazan 20 mg bid, amoxicillin 750 mg tid	76.6% (49/64)	90.6% (48/53)	9.4% (6/64)
Fan et al. [53] [2025]	China	252		vonoprazan 20 mg bid, amoxicillin 1000 mg bid	79.4% (200/252)	92.1% (197/214)	27.2% (67/246)
Peng et al. [54] [2025]	China	239		vonoprazan 20 mg bid, amoxicillin 1000 mg bid	91.6% (219/239)	91.5% (215/235)	11.7% (35/300)
Peng et al. [54] [2025]	China	247		vonoprazan 20 mg bid, amoxicillin 500 mg tid	87.0% (215/247)	86.8% (211/243)	13.0% (39/300)
All					86.0% (901/1048)	91.2% (888/974)	17.4% (201/1152)
***High-dose amoxicillin*** Chey et al. [55] [2022]	USA	265		vonoprazan 20 mg bid, amoxicillin 1000 mg tid	78.5% (208 /265)	81.2% (177/218)	29.9% (104/348)
Yang et al. [56] [2023]	China	200		vonoprazan 20 mg bid, amoxicillin 1000 mg tid	86% (172/200)	92.5% (172/186)	9.5% (17/200)
Peng et al. [57] [2023]	China	158		vonoprazan 20 mg bid, amoxicillin 750 mg qid	89.9% (142/158)	97.9% (142/145)	19.0% (30/158)
Hu et al. [58] [2023]	China	97		vonoprazan 20 mg bid, amoxicillin 1000 mg tid	88.6% (86/97)	95.5% (86/90)	16.67% (15/90)
Hu et al. [50] [2023]	China	55		vonoprazan 20 mg bid, amoxicillin 1000 mg tid	87.3% (48/55)	95.9% (47/49)	20.0% (11/55)
Jiang et al. [59] [2024]	China	200		vonoprazan 20 mg bid, amoxicillin 1000 mg tid	94.0% (188/200)	97.9% (188/192)	19.0% (38/200)
Huang et al. [60] [2024]	China	102		vonoprazan 20 mg bid, amoxicillin 1000 mg tid	92.2% (94/102)	93.9% (93/99)	13.7% (14/102)
Cheung et al. [61] [2024]	China	100		vonoprazan 20 mg bid, amoxicillin 1000 mg tid	96.0% (96/100)	96.7% (89/92)	39.0% (39/100)
Liu et al. [52] [2024]	China	64		vonoprazan 20 mg bid, amoxicillin 1000 mg tid	79.7% (51/64)	94.3% (50/53)	7.8% (5/64)
Lin et al. [62] [2024]	China	125		vonoprazan 20 mg bid, amoxicillin 1000 mg tid	91.20% (114/125)	93.39% (113/121)	36.1% (44/122)
Peng et al. [54] [2025]	China	251		vonoprazan 20 mg bid, amoxicillin 1000 mg tid	93.2% (234/251)	93.2% (233/250)	15.3% (46/300)
Song et al. [63] [2025]	China	209		vonoprazan 20 mg bid, amoxicillin 1000 mg tid	88.0% (184/209)	95.3% (181/190)	17.6% (36/204)
Yang et al. [47] [2025]	China	183		vonoprazan 20 mg bid, amoxicillin 1000 mg tid	84.2% (154/183)	96.8% (149/154)	9.84% (18/165)
All					88.2% (1771/2009)	93.5% (1720/1839)	19.8% (417/2108)
**14-day bismuth/regular-dose amoxicillin/high-dose vonoprazan triple therapy and bismuth/high-dose amoxicillin/high-dose vonoprazan triple therapy**							
***Regular-dose amoxicillin*** Liang et al. [66] [2024]	China		300	vonoprazan 20 mg bid, amoxicillin 750 mg tid, bismuth 220 mg bid	83.7% (251/300)	90.9% (251/276)	13.7% (41/300)
Qi et al. [67] [2025]	China		85	vonoprazan 20 mg bid, amoxicillin 1000 mg bid, bismuth 220 mg bid	89.4% (76/85)	92.7% (76/82)	4.9% (4/82)
All					84.9% (327/385)	91.3% (327/358)	11.8% (45/382)
***High-dose amoxicillin*** Hsu et al. [68] [2026]	Taiwan		120	vonoprazan 20 mg bid, amoxicillin 750 mg qid, bismuth 300 mg qid	95.8% (115/120)	98.4% (115/117)	10.0% (12/120)
All					95.8% (115/120)	98.4% (115/117)	10.0% (12/120)

Abbreviations: bid, twice a day; tid, three times a day; qid, four times a day.

### 2.3. Triple Therapies with Bismuth/Regular-Dose Amoxicillin/High-Dose Vonoprazan and Bismuth/High-Dose Amoxicillin/High-Dose Vonoprazan in the First-Line Treatment of H. pylori Infection

Table 2 summarizes eradication outcomes from randomized controlled trials published between 2014 and 2025 that evaluated 14-day first-line regimens combining bismuth with vonoprazan and either regular- or high-dose amoxicillin for *H. pylori* infection [66,67,68]. When data were pooled, the ITT eradication rates were 84.9% (327/385) for regular-dose amoxicillin regimens and 95.8% (115/120) for high-dose amoxicillin regimens. Corresponding PP eradication rates were 91.3% (327/358) and 98.4% (115/117), respectively, indicating particularly robust performance with the high-dose amoxicillin combination. Bismuth compounds are known to potentiate antimicrobial activity through multiple complementary mechanisms. They exert direct antibacterial effects by inhibiting critical enzymes—including urease, F1-ATPase, and alcohol dehydrogenase—and by interfering with bacterial oxidative defense systems, acid resistance mechanisms, and adhesion to gastric epithelial cells. Through these actions, bismuth enhances the activity of co-administered antibiotics, resulting in synergistic effects that improve overall eradication outcomes. Incorporating bismuth into triple regimens has been shown to substantially increase cure rates, particularly in infections involving resistant strains, where improvements of approximately 30–40% have been reported.

One randomized controlled trial [66] evaluated a 14-day regimen consisting of vonoprazan 20 mg twice daily, amoxicillin 750 mg three times daily, and bismuth 220 mg twice daily. This combination achieved ITT and PP eradication rates of 83.7% and 90.9%, respectively. Importantly, its efficacy was comparable to that of a bismuth-containing quadruple regimen composed of esomeprazole, bismuth, clarithromycin, and amoxicillin. More recently, a multicenter randomized trial enrolling 360 participants directly compared three first-line strategies: 14-day bismuth/high-dose amoxicillin/high-dose vonoprazan triple therapy, high-dose amoxicillin/high-dose vonoprazan dual therapy, and a conventional clarithromycin-based triple regimen (amoxicillin/clarithromycin/rabeprazole) [68]. The results demonstrated that the bismuth-containing triple therapy achieved significantly higher eradication rates than either the dual therapy or the clarithromycin-based triple therapy (98.4% vs. 87.2% and 87.1%, respectively).

### 2.4. Long-Term Consequences of Prolonged High-Dose Amoxicillin Exposure

Antibiotic administration is well recognized to influence intestinal microbial composition and reduce microbial diversity, thereby perturbing the gut ecosystem. Emerging evidence indicates that extended exposure to amoxicillin induces distinct changes in both the gut microbiome and the resistome in adults. One notable observation was a decline in the proportion of short-chain fatty acid (SCFA)–producing bacterial taxa that are generally associated with intestinal health. Reassuringly, these microbiological shifts were not permanent; longitudinal follow-up demonstrated that the overall community structure largely recovered to its pre-treatment state approximately nine months after therapy [69]. Comparable findings have been reported in the context of *H. pylori* eradication therapy. In a randomized controlled trial evaluating 10-day high-dose vonoprazan–amoxicillin (VA) dual therapy, a temporary reduction in microbial diversity and alterations in taxonomic composition were detected immediately following treatment. However, these disturbances were reversed by the scheduled post-eradication assessment. Although transient dysbiosis was observed shortly after completion of therapy, restoration of the gut microbial profile occurred within four weeks [70]. Taken together, available data suggest that vonoprazan-based amoxicillin dual therapy induces only modest and reversible changes in the gut microbiota and SCFA-producing bacteria, with no evidence of sustained long-term disruption.

## 3. Limitations

Several limitations should be acknowledged when interpreting the findings of this systematic review. First, most of the eligible studies were conducted in China, which may restrict the generalizability of the results to other regions with different antibiotic resistance patterns, genetic backgrounds affecting drug metabolism, and population characteristics. Second, the overall number of included trials was limited, particularly those evaluating 14-day regimens that combine regular- or high-dose amoxicillin with vonoprazan and bismuth. The relatively small evidence base for this specific triple therapy reduces the certainty of pooled estimates. Third, representation from Western countries was sparse, with only two studies originating from the United States and Portugal. Consequently, the external validity of the proposed therapeutic strategies in Western healthcare settings remains uncertain and warrants further investigation. Finally, although aggregated eradication rates and adverse event frequencies were calculated, a formal quantitative meta-analysis was not undertaken. Specifically, statistical assessments of between-study heterogeneity and potential publication bias were not performed, which may limit the robustness and interpretability of the overall conclusions.

## 4. Conclusions

Current international guidelines generally advocate tailoring *H. pylori* eradication regimens according to regional clarithromycin resistance rates. Nevertheless, an alternative strategy—employing a single susceptible antibiotic supported by strong acid suppression—has attracted increasing attention. Such an approach may streamline therapeutic protocols, enhance medication adherence, and reduce unnecessary exposure to multiple antibiotics while still addressing the challenge of antimicrobial resistance. In this systematic review, pooled PP eradication rates were 84.7% for regular-dose amoxicillin combined with high-dose PPI, 89.9% for high-dose amoxicillin with high-dose PPI, 93.5% for high-dose amoxicillin with high-dose vonoprazan, and 98.4% for bismuth plus high-dose amoxicillin and high-dose vonoprazan. These findings underscore the importance of achieving profound and sustained acid suppression, optimizing amoxicillin dosing, and leveraging the synergistic effects of bismuth to maximize treatment efficacy within amoxicillin-based mono-antibiotic frameworks. Despite these encouraging results, caution is warranted. Considerable variability exists among treatment protocols, and most available evidence originates from East Asian populations. Consequently, the external validity of these observations remains uncertain. Well-powered, rigorously designed randomized controlled trials conducted across diverse geographic and resistance settings are needed to establish the broader applicability of these regimens and to clarify their position in standard clinical practice.

## Figures and Tables

**Table 1:** Eradication rates of regular-dose amoxicillin-based mono-antibiotic therapies for the first-line treatment of *H. pylori* infection.

Author [Year]	Country	No. of Cases	Duration	Regimen	Eradication Rate
Rauws et al. [23] [1988]	Netherlands	22	4 weeks	amoxicillin 375 mg bid	22.7% (5/22)
Glupczynski et al. [24] [1988]	Belgium	45	8 days	amoxycillin suspension 20 mL (1000 g) bid	0% (0/45)
Oderda et al. [25] [1989]	Italy	42	4 weeks	amoxicillin 25 mg/kg bid	27% (8/30)

Abbreviation: bid, twice a day.

## Data Availability

No new data were created or analyzed in this study. Data sharing is not applicable.

## Supplementary Materials

The following supporting information can be downloaded at: https://www.mdpi.com/article/10.3390/microorganisms14030625/s1, PRISMA 2020 Checklist.
